# Galectins dysregulation: A way for cancer cells to invade and pervade

**DOI:** 10.32604/or.2022.026838

**Published:** 2023-01-12

**Authors:** MAHMOUD M. ABDELFATTAH, REHAM HELWA

**Affiliations:** 1 Molecular Cancer Biology Group, Zoology Department, Faculty of Science, Ain Shams University, Cairo, Egypt; 2 Faculty of Basic Science, King Salman International University, South Sinai, Egypt

**Keywords:** Galectins, Cancer, Metastasis, Erythrocytes, Invasion

## Abstract

Galectins are sticky molecules that bind to β-galactoside. Their interactions render them essential players in many cellular processes. The imbalance of galectin expression was reported in many diseases. In cancer, galectins interact with the extracellular matrix, evade the immune system, and potentially have broad interactions with blood components. In the last ten years, since 2010, we did focus on galectin research in different cancer types. Our findings showed an interaction between cancer cells and erythrocytes via galectin-4. Moreover, we found that upregulation of galectins was associated with lymph node metastasis in ovarian cancers. Hence, with this, we shortly review some important aspects of galectins and their potential importance in more profound understanding of cancer progression and the field of cancer biomarkers.

## Galectins are Potentially Supporting Cancer Cells to Invade and Metastasize via Interaction with Blood Components

**Figure 1:**
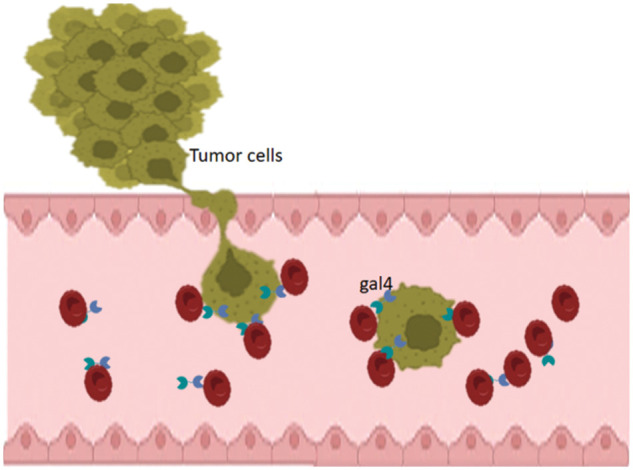
Schematic representation of the results of tumor-blood interaction. Tumor cells that exhibit upregulation of galectin-4 interact with red blood cells (RBCs). Galectins accumulate at the sites of attachment to the erythrocytes.

In 2017, an interaction between cancer cells and erythrocytes was reported and interpreted by galectin-4 interaction with the blood group antigen (Fig. 1). Displacement of galectin-4 to attachment points of cancer cells and erythrocytes was noticed. Also, we found in this article, a co-localization of galectin-4 and blood group antigen was seen using double fluorescent immunostaining. Moreover, a morphological deformation of red blood cells was seen to be associated with this interaction [1]. In this model, interacting cells were dividing without the presence of an attachment surface. In addition, developing lamellipodia/filopodia was noticed after interactions [1].

According to the structure of galectins, all surface/secreted galectins might interact with erythrocytes. Thus, many questions have been raised regarding the dysregulation of galectins in cancer. For instance, is the upregulation of galectins related to invasive cancers or lymph node metastasis? Thus, our group sought mRNA expression in many types of cancers, including AML [2,3], ovarian [4], endometrial, and breast (unpublished). Consistent with our hypothesis, in ovarian cancer, we found that galectin-9 might be a potential marker for lymph node metastasis [4].

## Supportive Biological Evidence and Functions Related to Galectins and Cancer

### Galectin family

**Figure 2:**
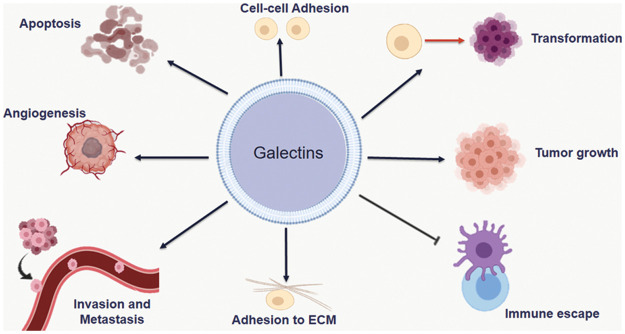
Different roles of Galectins in cancer. Galectins are involved in many events for cancer progression including; transformation, tumor growth, adhesion to extracellular matrux, immune escape, angiogenesis, invasion and metastasis.

Galectins are protein family that have a high affinity for binding to β-galactoside like N-acetyllactosamine via N-linked or O-linked glycosylation. Galectins are a structurally associated family containing at least one carbohydrate recognition domain (CRD) [5,6]. The CRD of this family is folded into a β-sandwich structure consisting of two stretched antiparallel β-sheets. Galectin’s ligand binds to the groove formed by β-sandwich [7]. Up to now, there are sixteen members of the galectins family. Depending on their structure, galectins are categorized into three different types: prototype, tandem repeat or chimera. Prototypical galectins (*LGALS*1, 2, 5, 7, 10, 11, 13, 14 and 15) contain one CRD that can dimerize. Tandem galectins (*LGALS*4, 6, 8, 9, and 12) are at least two CRD linked together by a small peptide domain. Galecin-3 is the only member that contains one CRD linked to the N-terminal non-lectin domain [8,9]. These structural aspects render them a key players in several cellular processes, as shown in Fig. 2.

### Subcellular localization

Galectins display a wide range of distribution (Table 1). They can be founded in the cytosol; and contribute to protein-protein interactions to mediate intracellular function or in extracellular places in which they depending on carbohydrate binding activities. Galectins family does not have a classical signal for transporting to ER–Golgi pathway. However, some members can be found in the extracellular matrix through non-classical secretion or an understood process [10,11]. Thus, galectins contribute to several functions such as the regulation of signaling pathways, such as cell-cell and cell-matrix interaction, pre-mRNA splicing, apoptosis, cell cycle, metastasis and immune response [12,13].

**Table 1:** Structural classification of galectins and their tissue and cell distributions

Galectin	Type	Cellular localization
Galectin-1	Prototype	Nucleus, cytosol and extracellular [14]
Galectin-2	Prototype	Nucleus [15]
Galectin-3	Chimera	Nucleus, cytosol and extracellular [16,17]
Galectin-4	Tandem repeat	Nucleus, Cytosol and extracellular [18,19]
Galectin-7	Prototype	Nucleus, Cytosol and extracellular [20,21]
Galectin-8	Tandem repeat	Nucleus, Cytosol and extracellular [22–24]
Galectin-9	Tandem repeat	Nucleus, Cytosol and extracellular [25,26]
Galectin-12	Tandem repeat	Nucleus, Cytosol [27]

## Galectins Dysregulation in Cancer

### Galectin-1

High expression of galectin-1 has been reported in several human tumors, such as lung, head and neck [28], colon [29,30], prostate [31], gastric [32], AML [2,3], and ovarian cancers [4]. Moreover, galectin-1 might be involved in tumor angiogenesis because endothelial cells and vascular smooth muscle express it [33]. Galectin-1 binds to neuropilin-1 and promotes VEGFR-2 phosphorylation, which increases cancer cells progression and migration [34–37]. In Colorectal Cancer cells, higher expression of galectin-1 is significantly correlated with metastasis recurrence and can activate the Wnt/β-catenin pathway [38]. It was reported that deletion of galectin-1 in pancreatic ductal adenocarcinoma leads to reduced metastasis levels and a significant increase in the survival, as a result of several mechanisms including improved T cell infiltration, decreased stroma activation, and decreased vascularization [39].

### Galectin-2

Our group did report overexpression of galectin-2 in monocytic AML. According to FAB classification, in peripheral blood, higher expression of galectin-2 was associated with M4 and M5. mRNA upregulation of galectin-2 was found to be associated with positive CD64, CD11c, and MHC class II [2]. In unpublished results, we could not detect galectin-2 mRNA in ovarian cancer tissues.

Galectin-2 attaches to human monocytes through CD14 and induces the expression of pro-inflammatory IFN-β, TNF-α, IL-6, IL12-p40, and decreased expression of pro-arteriogenic factors VEGF-A, MMP9, MMP2, PDGFB, and HGF [40].

### Galectin-3

Functions and expression of galectin-3 were repeatdly studied in several types of cancers.

Extracellular galectin-3 has been reported to stimulate the formation of new capillaries and enhance endothelial cell motility [41]. It interacts with N-glycans on αvβ3 integrin and trigger integrin signaling pathways that stimulate VEGF and bFGF angiogenic activity [42]. Overexpression of galectin-3 is associated with enhancing metastasis and poor prognosis in several types of cancers, including colon, thyroid, gastric, liver, pancreatic ductal adenocarcinoma, bladder, and breast cancer [43–46]. It has been revealed that galectin-3 binds to transmembrane mucin (MUC1) and triggers the clustering of MUC1 and exposures to smaller cell surface adhesion molecules, including E-cadherin [47]. Alternatively, the downregulation of *LGALS-*3 has been detected to be associated with the progression of cancer cells [48,49].

### Galectin-4

Galectin-4 has been reported in many cancers, and its expression is related to growth and progression. Most research on tumor-associated *LGALS*-4 levels reported changes at the mRNA level. Galectin-4 was elevated in hepatocellular carcinomas [50] and gastric cancer cells with increased metastatic potential [51]. Similarly, it was found in lung adenocarcinomas and was associated with cancer progression and metastasis [52]. Also, it was found to be elevated in breast cancer [53].

### Galectin-7

Galectin-7 is a prototype member of the galectins family containing a single CRD that forms a homodimer. Galectin-7 is produced in the cytoplasm, accumulates in the nucleus or cytosol, then secreted to the outer plasma membrane or extracellular matrix [54,55]. Galectin-7 has a reverse effect on tumor growth and progression. It may have an apoptotic effect on certain types of tumors. However, it can promote the development of others. It was reported that a high amount of galectin-7 was found after UVB irradiation of the skin which induces apoptosis and has a significant effect on keratinocyte [56]. Moreover, the ectopic expression of galectin-7 has an apoptotic effect in the human colon carcinoma cell line DLD-1 [57]. In gastric cancer, there was significant statistical correlation between low levels of galectin-7 in tumor tissues compared with corresponding normal tissues [58]. Galectin-7 contributes to apoptosis through interaction with the anti-apoptotic factor Bcl-2 in a carbohydrate-independent manner [59].

Conversely, galectin-7 can enhance invasion activity in human oral squamous cell carcinoma cells, via several mechanisms, including activation of ERK and JNK signaling and the induction of MMP-2 and MMP-9 [60]. Similarly, overexpression of galectin-7 in breast cancer is sufficient to promote metastasis to the bone and lung-related with metastasis of lymph node axillary. Moreover, it can increase the resistance to apoptosis in melanoma cells [61,62].

### Galectin-8

Galectin-8 is a potential tumor marker of papillary thyroid cancer, since it was detected in the majority of thyroid cancers, is undetectable in normal thyroid [63]. Also, increased concentration of galectin-8 has been reported in lung cancer and correlated to cancer progression and metastasis [64]. Galectin-8 has been reported to bind to CD166 and stimulate angiogenesis [65].

The expression of galectin-8 is significantly associated with relapse-free survival in patients with squamous cancer cells in cervical cancer [66].

Similarly, galectin-8 was found to stimulate the progression and metastasis in prostate cancer. In addition, it has been shown that galectin-8 can bind directly to K-Ras and moderate cell proliferation and migration in lung and pancreatic cancer cells [67].

### Galectin-9

Galectin-9 was previously described as a protein that is differentially expressed in immune cells and known to be involved in several biological functions, such as regulation of cell adhesion, migration, cell polarity, chemotaxis, proliferation, apoptosis, and differentiation [68,69]. T-cell immunoglobulin and mucin domain-containing molecule 3 (TIM-3) is a transmembrane protein that acts as a receptor for galectin-9 [70]. TIM-3 is expressed over the cell surface of leukemic stem cells (LSCs) in all types of acute myeloid except the subtype M-3, and is not expressed in normal hematopoietic stem cells (HSCs) [71].

The binding of galectin-9/TIM-3 is critical for transformed normal HSCs to LSCs and self-renewal of LSCs, because they induce the phosphorylation of both NF-κB and β-catenin signaling through stimulating phosphorylation of ERK and AKT. Neutralized galectin-9 significantly reduces the self-renewing of LSCs and increases the apoptosis rate of LSCs [72]. In AML cells, TIM-3 and its ligand galectin-9 stimulate the activation of the PI3K/mTOR pathway. Also, it induces the expression of HIF-1α and VEGF [73].

Galectin-9-TIM-3 interaction regulates the immunosuppression by promoting apoptosis of T-cells and inhibits the activity of natural killer NK cells [74]. It also reported that Galectin-9/TIM-3 induced apoptosis of Th1 and improved differentiation of monocyte towards M2 macrophage [75]. Galectin-9 acts in two phasic patterns on the activity of T-cells depending on its concentration and not depending on TIM-3 [76].

### Galectin-10

Galectin-10 is also known as the Charcot-Leyden crystal (CLCP). This glycan binding protein typically forms bi-pyramidal hexagonal crystals, identified as the Charcot-Leyden crystals [77]. Galectin-10 is found in various granules of eosinophils [78,79]. Galectin-10 is highly expressed in the bone marrow, which play significant role in lymph cell maturation [80]. Galectin-10 was decreased in ovarian cancer cells compared with normal cells, and a higher level of expression is associated with a better OS [81].

### Galectin-12

Galectin-12 has been described as having a significant role in cell cycle activity. High expression of galectin-12 is associated with apoptosis in adipose tissue [82]. Also, upregulated galectin-12 induces apoptosis through the cell cycle arrest at the G1 phase and inhibits cell proliferation [83]. These results suggest the effect of galectin-12 in pro-apoptotic function and its role in cellular homeostasis. Galectin-12 is also expressed in macrophages. Deletion of galectin-12 reduced the activity of phagocytosis against *Escherichia coli* and lowered the concentration of nitric oxide. Silencing of galectin-12 induces differentiation of macrophages into the M2 subtype and decreases expression levels of the M1subtype, besides reduced expression levels of numerous M1 pro-inflammatory cytokines [84].

### Galectin-13

Galectin-13 is a prototype member of the galectins family, which was first isolated from the human placenta [85]. It is expressed in the spleen, kidney, and bladder, as well as in liver cancer, malignant melanoma, and neurogenic tumors [86]. Like other galectins, galectin-13 is expressed in the cytoplasm and can be secreted to the extracellular matrix through a non-classical transport pathway. Galectin-13 can promote apoptosis of activated T-cells and control immune tolerance between maternal and fetal tissues [87]. It was found that galectin-13 can influence neutrophils by reducing the apoptosis rate. Moreover, Galectin-13 increases the expression level of PD-L1, HGF, TNF-α, reactive ROS, and MMP-9 [88].

### Galectin-14

Galectin-14 is a prototype galectin with significant homology to galectin-13, which is also known as Placental Protein 13 (PPL 13) [89]. Higher expression of Galectin-14 is detected in epithelial ovarian cancer compared to normal tissues and is significantly associated with decreased survival of OC [90].

Galectin-14 was identified in placental tissue [91] and, together with galectins-13 and -14 was found to be elevated in distinguished trophoblast cells to confer immunotolerance at the maternal–fetal interface placenta [92].

## Research Perspective

There are still many question marks around galectins in cancer. Interactions of galectins with all blood components are still ambiguous. These interactions could be key players in cancer progression and metastasis. Several research projects have to be assigned to confirm the interactions of galectins and explore their pathophysiological role. No doubt that resolving the exact mechanism will unravel many events of cancer progression.

Thus, these molecules are of great interest to be added to the biomarkers panel of several cancers for prognosis or detection. In addition, they could be therapeutic targets to prevent invasion and metastasis.

## Data Availability

Not applicable.
